# Serum Metabolic Profiles of the Tryptophan-Kynurenine Pathway in the high risk subjects of major depressive disorder

**DOI:** 10.1038/s41598-020-58806-w

**Published:** 2020-02-06

**Authors:** Masashi Sakurai, Yasuko Yamamoto, Noriyo Kanayama, Masaya Hasegawa, Akihiro Mouri, Masao Takemura, Hidetoshi Matsunami, Tomoya Miyauchi, Tatsuya Tokura, Hiroyuki Kimura, Mikiko Ito, Eri Umemura, Aiji Sato (Boku), Wataru Nagashima, Takashi Tonoike, Kenichi Kurita, Norio Ozaki, Toshitaka Nabeshima, Kuniaki Saito

**Affiliations:** 1 Human Health Sciences, Graduate School of Medicine and Faculty of Medicine, Kyoto University, Kyoto, 606-8507 Japan; 2 Department of Disease Control and Prevention, Fujita Health University Graduate School of Health Sciences, Toyoake, 470-1192 Japan; 3 Department of Regulatory Science, Fujita Health University Graduate School of Health Sciences, Toyoake, 470-1192 Japan; 4 Japanese Drug Organization of Appropriate Use and Research, Nagoya, 468-0069 Japan; 5 Matsunami Research Park, Gifu, 501-6062 Japan; 6 Department of Psychiatry, Nagoya University, Graduate School of Medicine, Nagoya, 466-8550 Japan; 7 Department of Oral and Maxillofacial, Surgery, School of Dentistry, Aichi Gakuin University, Nagoya, 470-0195 Japan; 8 Department of Anesthesiology, Aichi Gakuin, University, Nagoya, 470-0195 Japan; 9 Department of Psychopathology and Psychotherapy/Center for Student Counseling, Nagoya University, Graduate School of Medicine, Nagoya, 466-8550 Japan; 10 Faculty of Psychological and Physical Sciences, Health Service Center, Aichi Gakuin University, Nisshin, Japan; 11 Advanced Diagnostic System Research Laboratory, Fujita Health University Graduate School of Health Sciences, Toyoake, 470-1192 Japan

**Keywords:** Predictive markers, Depression

## Abstract

Previous reports have shown that during chronic inflammation, the tryptophan (TRP)-kynurenine (KYN) pathway plays a pivotal role in the onset of depression. The aim of this study was to investigate the characteristics of the serum TRP-KYN pathway metabolite profile in high-risk subjects of major depressive disorder (HRMDD) defined by depression scores. The concentrations of TRP-KYN pathway metabolites {TRP, KYN, 3-hydroxyanthranilic acid (3HAA), 3-hydroxykynurenine (3HK), kynurenic acid (KYNA) and anthranilic acid (AA)} were assessed in serum from HRMDD, chronic pain disorder patients and healthy controls. In serum from HRMDD, elevated levels of AA and decreased levels of TRP were observed, but the levels of other metabolites were not changed. Furthermore, the change in the AA_2nd_/AA_1st_ ratio in subjects who progressed from a health. y state to a depressive state was correlated with an increase in the CES-D score. The level of IL-1 receptor antagonist (IL-1RA) was negatively correlated with that of AA. Interestingly, we confirmed AA as a possible biomarker for depression-related symptoms, since the metabolite profiles in the chronic pain disorder group and chronic unpredictable mild stress model mice were similar to those in the HRMDD. These results suggest that AA may be an effective marker for HRMDD.

## Introduction

More than 300 million people suffered from major depressive disorder (MDD) in 2017, and the number is increasing year by year^1^. Several theories of MDD onset have been proposed^2^. The monoamine hypothesis, the chronic inflammation hypothesis, and the abnormalities in the hypothalamus-pituitary-adrenal (HPA) system hypothesis are the predominant hypotheses regarding the pathogenesis of MDD^3^. Among these hypotheses, the chronic inflammation hypothesis is closely associated with the kynurenine (KYN) pathway^4^. The KYN pathway is one of several tryptophan (TRP) metabolism pathways, and it is the main pathway involved in TRP metabolism^5,6^. Inflammatory cytokines such as IFN-γ induce the expression of indoleamine 2,3-dioxygenase (IDO1), which is a limiting enzyme in the KYN pathway and is involved in one of the late steps^7,8^. Various lines of evidence suggest that KYN pathway activity is involved in the symptoms of MDD^9–11^.

The KYN pathway produces various metabolites, such as KYN, 3-hydroxykynurenine (3HK), anthranilic acid (AA), kynurenic acid (KYNA), 3-hydroxyanthranilic acid (3HAA) and quinolinic acid (QUIN). These metabolites have been reported to have various physiological activities. For example, 3HK and QUIN are known to cause neurotoxicity due to their active oxygen production and their agonistic activity via NMDA-type glutamate receptors. Thus, metabolic changes in the KYN pathway may cause various biological responses in depression. Increasing evidence has been gathered regarding the relationship between the activation of the KYN pathway and the onset of depression. Increased inflammation and increased IDO1 activity induce depressive-like behaviour in animal models^12,13^. In addition, learning and the speed of processing in females with MDD are associated with the serum level of KYN and the KYN/TRP ratio^14^. Furthermore, increases in serum concentrations of KYN and 3HK and in the KYN/TRP and 3-HK/KYNA ratios are much greater in depressive patients with hepatitis C virus than nondepressed patients following therapy^15^. Darlington *et al*. have shown that the plasma concentrations of AA in depressive patients are higher than those in healthy controls^16^. Another study reported increased serum AA levels in patients with schizophrenia^17^.

We speculate from these data that TRP metabolites are useful biomarkers for the realization of pre-emptive medicine in depressive symptoms. Pre-emptive medicine is a new concept in preventive medicine. It involves predicting disease before onset and preventing and delaying onset. To facilitate pre-emptive medical care, it is important to develop effective biomarkers for the early diagnosis of disease. However, there are currently no effective established biomarkers that can be used to diagnose depressive symptoms in the early stages. Therefore, we examined the change in TRP metabolites in high-risk subjects of MDD (HRMDD). By comparing the change in the metabolites in the subjects who progressed from a healthy to a depressive state, we investigated whether the TRP metabolite profile is useful for the detection of depressive symptoms. We also compared the changes in the concentrations of metabolites in the healthy controls with those of patients in the chronic pain disorder group to determine the similarities and differences in the profiles.

## Results

### Differences in the concentrations of TRP metabolites between the HRMDD and control groups

To examine the changes in the concentrations of TRP metabolites in the HRMDD and healthy control groups, the TRP metabolites TRP, KYN, 3HAA, KYNA, 3HK and AA were measured by high performance liquid chromatography (HPLC). The details regarding the age, sex, depression scores and values of some of the clinical parameters of the subjects are summarized in Table 1.

**Table 1:** Characteristics of the healthy control and the high risk subjects of MDD (HRMDD) groups.

	Male		Female	
	Healthy control	HRMDD	Healthy control	HRMDD
Number of subjects	38	42	21	19
Age	46.1 (7.4)	46.0 (6.6)	43.3 (7.3)	46.5 (8.1)
BMI	21.8 (1.8)	24.0 (3.3)	20.4 (1.6)	20.7 (2.5)
SBP (mmHg)	115.9 (9.8)	125.2 (17.6)	108.7 (11.3)	112.4 (12.0)
DBP (mmHg)	73.1 (6.9)	79.6 (11.9)	67.9 (7.2)	69.7 (7.8)
LDL (mg/dL)	112.7 (14.7)	128.2 (32.7)	97.9 (20.4)	113.2 (31.6)
HDL (mg/dL)	52.7 (7.9)	51.5 (12.7)	65.2 (11.9)	63.2 (11.9)
TG (mg/dL)	87.8 (38.5)	115.5 (68.5)	69.6 (25.3)	74.3 (46.6)
FBS (mg/dL)	92.8 (8.0)	99.8 (29.8)	89.8 (5.6)	94.9 (13.7)
GHQ28 Score	2.8 (2.6)	14.2 (4.5)	3.0 (2.2)	14.4 (3.0)
CES-D Score	6.7 (4.3)	33.0 (8.3)	8.6 (3.7)	32.1 (8.9)

BMI: body mass index; SBP: systolic blood pressure; DBP: diastolic blood pressure; LDL: low-density lipoprotein; HDL: high-density lipoprotein; TG: triglyceride; FBS: fasting blood sugar.

**Figure 1:**
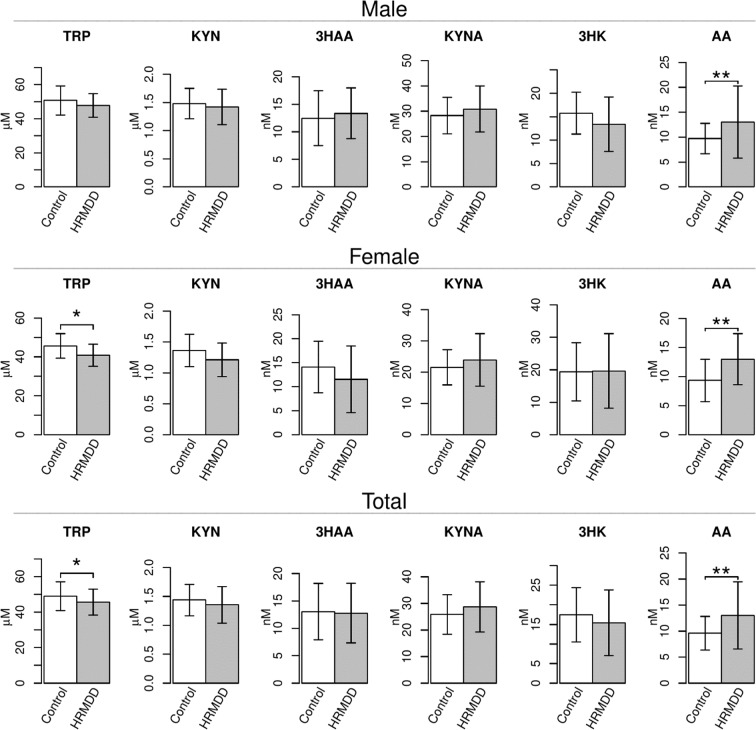
Differences in the concentrations of TRP metabolites between the healthy control and high risk subjects of MDD (HRMDD) groups. The TRP metabolites, such as TRP, kynurenine (KYN), 3-hydroxyanthranilic acid (3HAA), kynurenic acid (KYNA), 3-hydroxykynurenine (3HK), and anthranilic acid (AA), were examined by HPLC in the healthy control (n = 59, ) and HRMDD groups (n = 61, ). The obtained values are expressed as the mean ± SD. *P < 0.05, **P < 0.01; significant difference in values as determined by Welch’s t test.

In both males and females, the concentration of AA was significantly higher in the HRMDD group than the healthy control group (Fig. 1). The concentration of TRP in females of HRMDD group was significantly lower than that of healthy control group. We calculated the ratios of the metabolites to estimate the activity of the enzymes in the metabolic pathway. The AA/KYN ratios were significantly higher in both male and female HRMDD patients than healthy control subjects (Table 2). The 3HAA/3HK and 3HAA/AA ratios were significantly lower in female HRMDD patients, but the KYNA/KYN ratio was significantly higher in male HRMDD patients than the corresponding healthy control subjects. These data suggest that TRP metabolites could be useful markers for depressive symptoms.

**Table 2:** Concentrations of tryptophan metabolites and relative ratios of the metabolites in the healthy control and the high risk subjects of MDD (HRMDD) group.

	Male		Female	
	Healthy control	HRMDD	Healthy control	HRMDD
Kyn (μM)	1.48 (0.27)	1.42 (0.31)	1.36 (0.26)	1.21 (0.27)
Trp (μM)	50.7 (8.54)	47.8 (6.96)	45.6 (6.29)	40.8 (5.66)*
3HAA (nM)	12.5 (4.98)	13.3 (4.60)	14.1 (5.37)	11.5 (6.94)
KYNA (nM)	28.2 (7.24)	30.8 (9.15)	21.5 (5.59)	23.9 (8.40)
AA (nM)	9.73 (3.03)	13.0 (7.24)**	9.36 (3.65)	13.0 (4.38)**
3HK (nM)	15.8 (4.46)	13.4 (5.82)	19.4 (8.96)	19.7 (11.5)
Kyn/Trp	0.0297 (0.00593)	0.0304 (0.00447)	0.0300 (0.00647)	0.0300 (0.00477)
KYNA/Kyn	0.0159 (0.00373)	0.0192 (0.00612)*	0.0152 (0.00399)	0.0178 (0.00495)
AA/Kyn	0.0060 (0.00205)	0.0099 (0.00566)**	0.0067 (0.00258)	0.0115 (0.00404)***
3HK/Kyn	0.0102 (0.00225)	0.0088 (0.00360)	0.0135 (0.00640)	0.0148 (0.00792)
3HAA/3HK	0.946 (0.274)	1.17 (0.465)	1.04 (0.270)	0.651 (0.434)*
3HAA/AA	1.27 (0.701)	1.00 (0.552)	1.37 (0.517)	0.707 (0.369)***

### Differences in TRP metabolite and cytokine concentrations changes in subjects who progressed from a healthy state to a depressive state

We observed the annual changes in TRP metabolism and Centre for Epidemiological Studies Depression scale (CES-D)^18^ scores in individuals. The group of subjects who progressed from a healthy state to HRMDD was selected according to the following criteria: a CES-D score for the first test (CES-D_1st_) < 16, a CES-D score for the second test (CES-D_2nd_) ≥ 16 and a value of CES-D_2nd_ - CES-D_1st_ > 10. The age-matched controls were selected according to the following criteria: CES-D scores for the first and second tests <8. The details regarding the age, sex, BMI and CES-D scores of the subjects are summarized in Table 3.

**Table 3:** Characteristics of healthy control and the subjects who progressed from a healthy state to a depressive state (high risk subjects of MDD, HRMDD).

		Male		Female	
		Healthy control	HRMDD	Healthy control	HRMDD
Number of subjects		34	17	32	16
Age		50.1 (7.1)	49.8 (7.2)	44.0 (8.1)	43.9 (7.7)
BMI		23.9 (3.0)	23.6 (2.8)	21.2 (2.9)	19.4 (2.0)
CES-D Score	1st	4.5 (2.1)	9.9 (3.4)	3.0 (2.4)	9.7 (3.6)
	2nd	4.4 (1.8)	30.2 (11.6)	3.6 (2.4)	26.5 (7.0)

**Figure 2:**
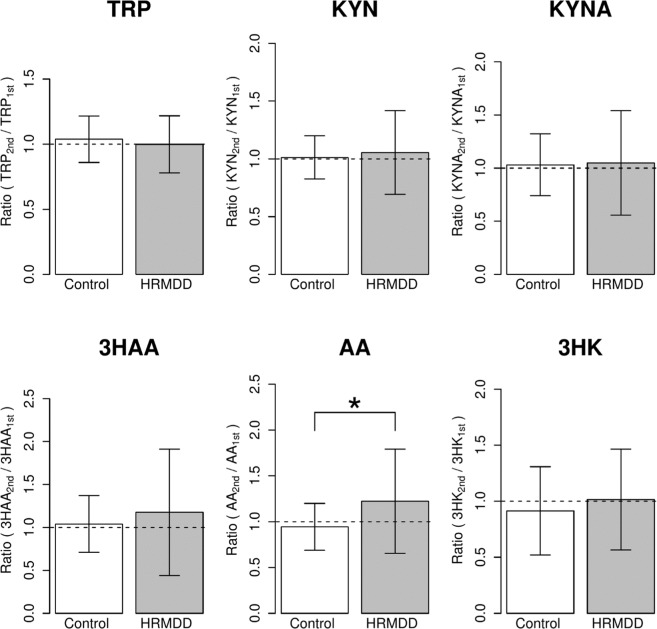
Differences in the ratios of TRP metabolites in the healthy control and subjects who progressed from a healthy state to a depressive state (high risk subjects of MDD, HRMDD). Changes in the ratios of TRP metabolites (X_2nd_/X_1st_) in the healthy control (n = 66, ) and HRMDD groups (n = 33, ). TRP metabolites, such as TRP, KYN, 3HAA, KYNA, 3HK, and AA, were examined by HPLC. The obtained values are expressed as the mean ± SD. *P < 0.05; significant difference in values between the healthy control and HRMDD groups as determined by Welch’s t test.

To evaluate the changes for individual subjects, we calculated the change ratios for each metabolite as X_2nd_/X_1st_ (Supplemental Table 1). The changes in the AA_2nd_/AA_1st_ ratio in the HRMDD group were significantly greater than those in the control group (Fig. 2).

**Figure 3:**
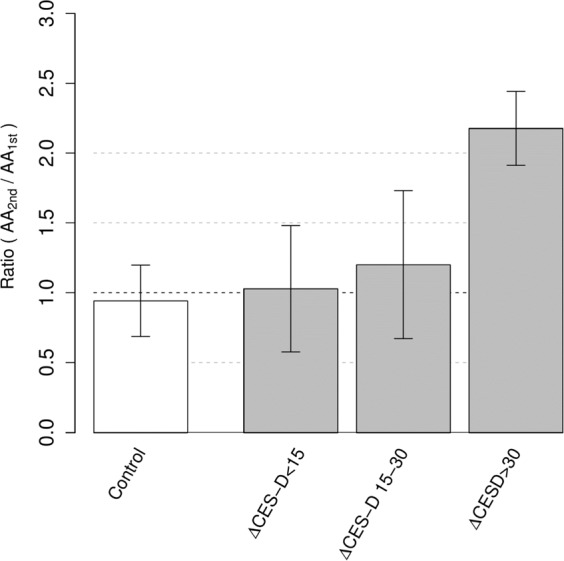
Relationship between the ratio of anthranilic acid (AA_2nd_/AA_1st_) and the difference in the CES-D scores (ΔCES-D). AA_2nd_/AA_1st_ shows the ratio of AA levels (AA_2nd_/AA_1st_). ΔCES-D represents the difference in the CES-D scores from the first test and the second test: ΔCES-D = CES-D_2nd_ - CES-D_1st_. Healthy control (n = 66, ), HRMDD groups (n = 33, ).

To investigate the relationship between changes in the ratios of TRP metabolites and the increase in the CES-D score, which indicates the progression of depression, we calculated the increase in the CES-D score according to the formula ΔCES-D = CES-D_2nd_ - CES-D_1st_. ΔCES-D was correlated with the change in the AA_2nd_/AA_1st_ ratio (Fig. 3). In the subjects with a low ΔCES-D value (<15), the change in the AA_2nd_/AA_1st_ ratio was approximately 1, and the distribution of the change ratios was similar to that observed in the control subjects; however, in subjects with a large ΔCES-D value (>30), the AA_2nd_/AA_1st_ ratio was much greater than 1.

**Figure 4:**
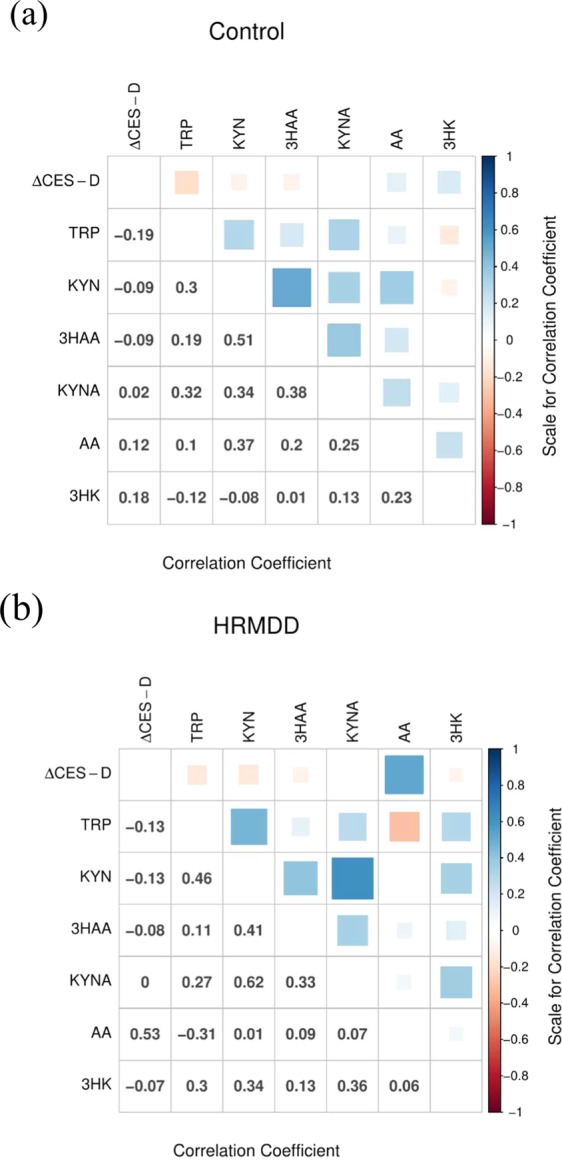
Correlation matrix showing the difference in the CES-D scores (ΔCES-D) and the changes in the ratios of TRP metabolites. Correlation matrix showing the difference in the CES-D scores (ΔCES-D) and the changes in the ratios of TRP metabolites for healthy controls (n = 66) (**a**) and subjects who progressed from a healthy state to a depressive state (high-risk MDD subjects, HRMDD; n = 33). (**b**) In the upper triangular matrix, the blue colour indicates a high positive correlation, and the red colour indicates a high negative correlation. In the lower triangular matrix, the number in each cell represents the correlation coefficient (Pearson’s product moment correlation coefficient).

In the correlation matrix that showed the relationship between the ΔCES-D values and the changes in the ratios of TRP metabolites, only the AA_2nd_/AA_1st_ ratio was correlated with ΔCES-D in the HRMDD group (Fig. 4). The correlation coefficient between ΔCES-D and the AA_2nd_/AA_1st_ ratio in the HRMDD group was 0.53 (P < 0.005). The changes in the TRP and AA ratios in the HRMDD group showed a weak negative correlation, whereas the changes in other ratios in both groups showed weak or moderate correlations with the change in the ratio of TRP, which is upstream of the KYN pathway (Fig. 4). These results suggest that AA is a good biomarker for HRMDD.

**Figure 5:**
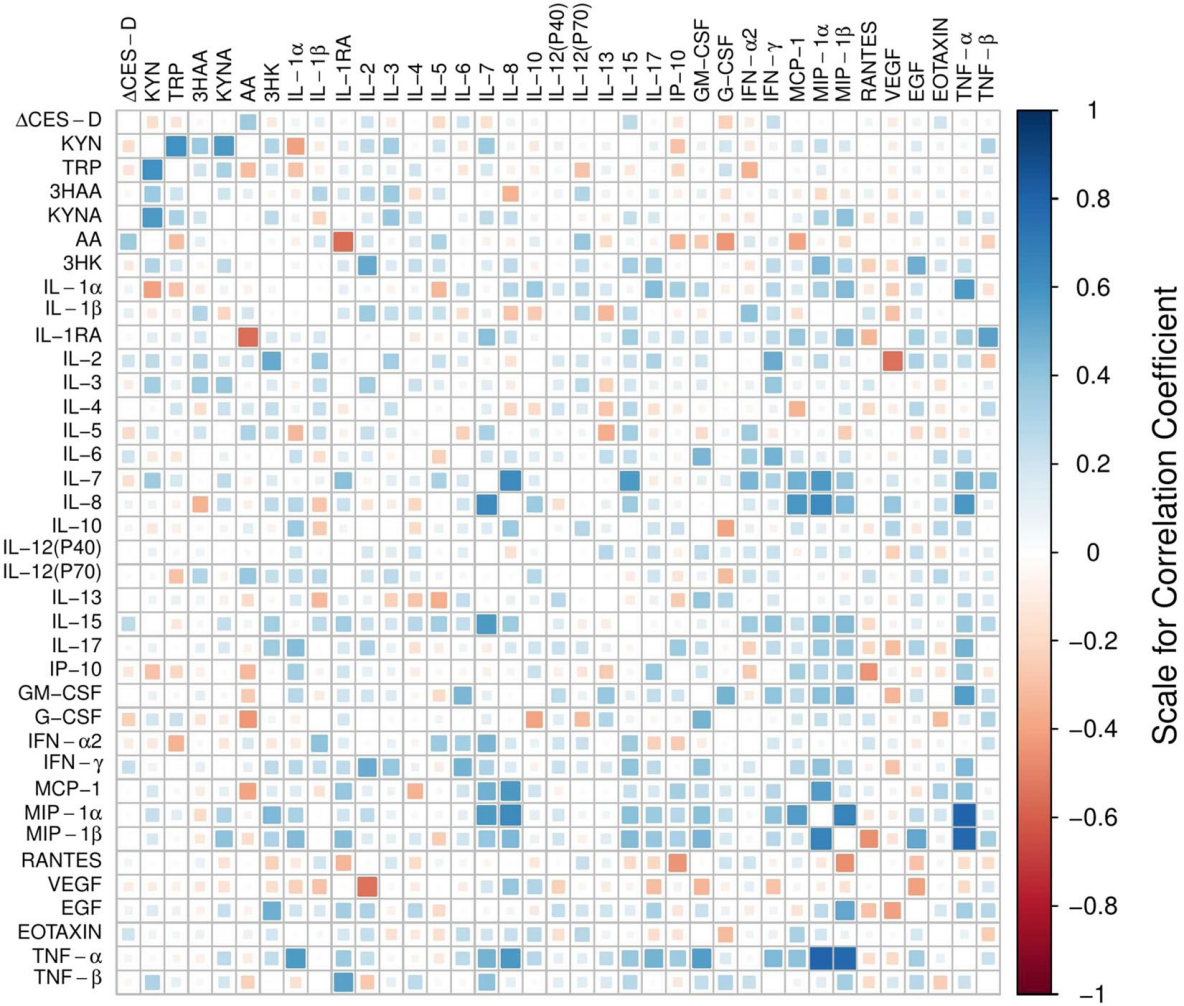
The correlation matrix showing the difference in the CES-D scores and the changes in the concentrations of TRP metabolites and cytokines in subjects who progressed from a healthy state to a depressive state. The blue colour indicates a high positive correlation, and the red colour indicates a high negative correlation. The correlation coefficients are calculated by Pearson’s product moment correlation coefficient (n = 33).

Because cytokine levels affect TRP metabolism, the association of TRP metabolites with cytokines was investigated in subjects who progressed from a healthy state (X_1st_) to a depressive state (X_2nd_) (Supplemental Table 2). The correlation matrix between ΔCES-D and the levels of cytokines was examined. IFN-γ and IL-6 correlated slightly with the CES-D score (Fig. 5 and Supplemental Table 3). The correlation matrix between the change in the TRP metabolites and the levels of cytokines showed that the changes in G-CSF, IL-1RA, IL12-P70 and MCP-1 levels were moderately correlated with the change in AA (Fig. 5). The correlation coefficients were as follows: G-CSF (r = −0.43, p < 0.05), IL-1RA (r = -0.56, p < 0.005), IL-12 p70 (r = 0.37, p < 0.05), and MCP-1 (r = -0.40, p < 0.05). This correlation matrix was calculated according to the difference in the concentration of X_2nd_ and X_1st_ instead of the ratio between the two.

### Differences in the concentrations of TRP metabolites between the chronic pain disorder and healthy control groups

**Figure 6:**
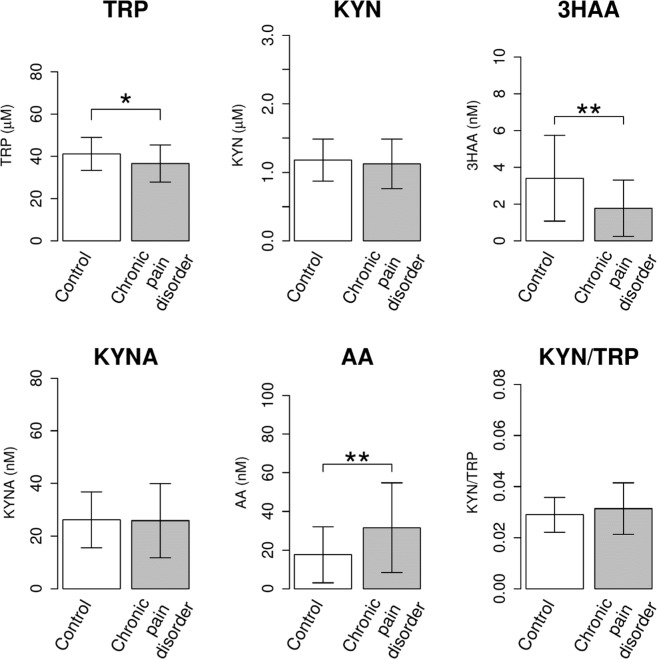
Differences in the concentrations of TRP metabolites in the healthy control and chronic pain disorder groups. The concentrations of TRP metabolites, such as TRP, KYN, 3HAA, KYNA and AA, in the healthy control (n = 42) and chronic pain disorder groups (n = 48) were examined by HPLC. The obtained values are expressed as the mean ± SD. *P < 0.05, **P < 0.01; significant difference in values as determined by Welch’s t test.

Chronic pain causes depression, and the onset of pain and depression has been closely linked to inflammation^19–21^. To expand our hypothesis, we examined the changes in the concentrations of TRP metabolites in patients with chronic pain disorders {burning mouth syndrome (BMS) and atypical odontalgia (AO)} affecting the orofacial region. Among the chronic pain disorder patients, 4 subjects were diagnosed with MDD, and the mean ± standard deviation (SD) of the Hamilton Depression Rating Scale (HDRS)^47^ score was 6.69 ± 5.10. BMS/AO patients had increased levels of AA, whereas the concentrations of TRP and 3HAA in the pain disorder group were significantly lower than those in the control group (Fig. 6). The HDRS high-score group (${\rm{HDRS}}\ge 10$) showed higher AA levels than the HDRS low-score group (${\rm{HDRS}} < 10$), but the difference was not significant (Supplemental Fig. 1). These results indicate that AA may be a good biomarker for HRMDD since chronic pain is strongly associated with HRMDD^19^.

### AA levels are increased in a chronic unpredictable mild stress (CUMS) mouse model

**Figure 7:**
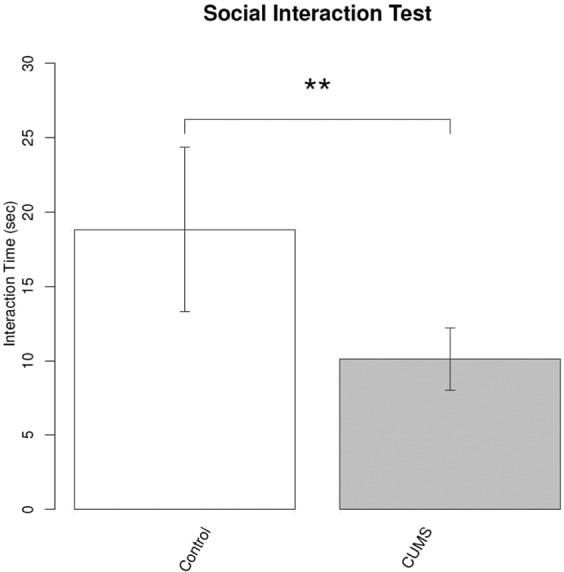
Differences in the interaction time in control mice and chronic unpredictable mild stress mice. A social interaction test was performed to confirm the stress in the chronic unpredictable mild stress model. The interaction time of stressed mice was shorter than that of control mice. **P < 0.01; significant difference in values as determined by Welch’s t test (n = 12 mice per group).

**Figure 8:**
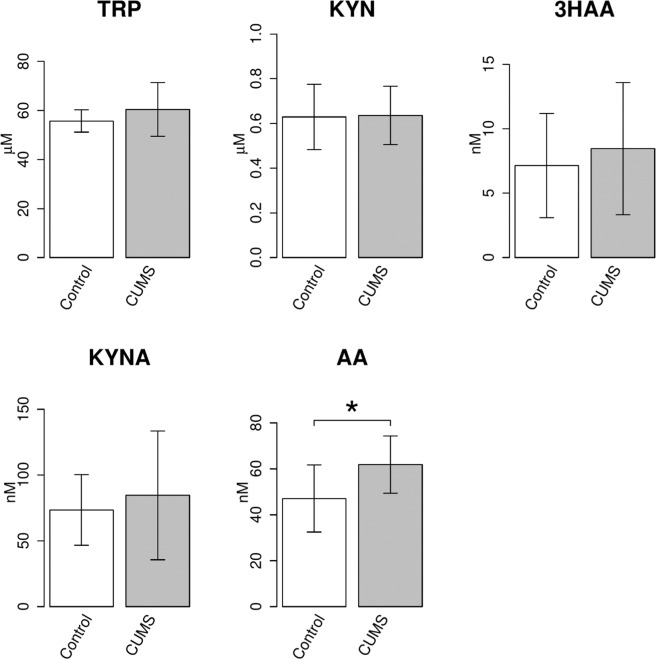
Increase in the levels of AA in chronic unpredictable mild stress (CUMS) mice. The concentrations of TRP metabolites, such as TRP, KYN, 3HAA, KYNA and AA, were examined by HPLC. The obtained values are expressed as the mean ± SD. *P < 0.05; significant difference in values as determined by Welch’s t test (n = 12 mice per group).

To confirm the serum tryptophan metabolite changes, we investigated whether the CUMS model mice showed increased levels of AA. The social interaction time with an unknown partner in CUMS mice was lower than that in control mice (Fig. 7). CUMS induced depression-like behaviours in mice. Among the TRP metabolites, AA levels were increased in CUMS mouse serum compared to control mouse serum (Fig. 8).

## Discussion

Links between various inflammatory diseases, such as hepatitis C (HC), autoimmune disorders, cardiovascular disease, diabetes and cancer, and the development of depression have been reported^19,22,23^. Interestingly, it has been reported that chronic inflammation is closely related to the onset of depression^24,25^. For example, proinflammatory cytokines, such as IL-6, IL-1β and TNFα, decrease hippocampal neurogenesis, which is related to depression^26^, and the secretion or production of these cytokines is increased in stressed individuals and in patients with depression^27–29^. In fact, in patients with MDD, the level of IL-6 in cerebrospinal fluid (CSF) is higher than that in healthy controls^30^.

It is possible that chronic inflammation (neuroinflammation) causes an increase in inflammatory cytokine levels and results in the activation of the KYN pathway^31,32^. Interestingly, the KYN pathway is more highly activated in patients with various psychiatric disorders than healthy subjects. Changes in the concentrations of metabolites and enzymatic activity observed in the post-mortem brains of patients with schizophrenia and an analysis of CSF produced the same conclusion^33,34^. Furthermore, the activation of the KYN pathway in patients with bipolar mood disorder is supported by the possibility of an increased risk of the disease due to changes in the expression of a gene encoding a molecule that participates in the KYN pathway^35^. Moreover, KYN pathway dysregulation has also been observed in MDD^36^.

The results of the present study show that the AA concentration in the HRMDD group was higher than that in the healthy control group. The increase in the AA level is consistent with that in other studies showing a relationship between AA levels and mental disorders. In patients with chronic HC, the serum AA concentration is increased compared with that measured at baseline^37^. Furthermore, in patients with depression^16^, schizophrenia^17^ and preclinical Alzheimer’s disease (AD)^38^, increased AA levels have also been observed. However, the biological function of AA has not yet been revealed. Although further study is needed to elucidate the relationship between the onset of depression and the change in the concentration of AA, our data show that AA may be a useful biomarker to detect the early stages of depression.

In the pathogenesis of inflammation-related depression, it is important to shift tryptophan metabolism from serotonin synthesis to kynurenine synthesis. In our unpublished data, serotonin levels in the serum did not change in response to any type of stress in mice because platelets produce much serotonin. Therefore, in the present study, we did not evaluate the serum serotonin levels.

Furthermore, our data show that the changes in G-CSF, IL-1RA, IL-12 p70 and MCP-1 levels were moderately correlated with the changes in the concentration of AA. In particular, IL-1RA showed a high correlation with AA in HRMDD patients. IL-1RA functions as a natural antagonist for both IL-1α and IL-1β and is produced by monocytes and macrophages^39^. Several studies have shown that IL-1RA, which is constitutively expressed in the brain, has a neuroprotective function^40^. Additionally, the intracerebroventricular administration of IL-1RA prevents the development of behavioural deficits in a learned helplessness model of depression^41^. In patients with MDD, serum IL-1RA levels were found to be higher than those in healthy controls^42^. In addition, in maternal depression, the symptoms of depression were found to be associated with reduced levels of IL-1RA^43^.

Interestingly, our data show that an increase in the AA concentration is associated with decreased levels of IL-1RA in HRMDD. These data suggest that HRMDD may be characterized by an imbalance of anti-inflammatory factors.

We further investigated the TRP profiles in the disease groups to determine whether there is a positive correlation between the changes in the concentrations of TRP metabolites and clinical symptoms. In the chronic pain disorder group, a reduced concentration of TRP and an increased concentration of AA confirmed the results for HRMDD. Furthermore, the CUMS mouse model showed higher serum AA levels.

Our data suggest that it is important to investigate HRMDD to establish biomarkers to detect the early stages of depression. Overall, these findings suggest that AA may be a sensitive biomarker that could be used to detect HRMDD. Monitoring TRP metabolites may be useful for determining disease progression in depression. Furthermore, it may provide a benefit in terms of the establishment of personalized treatment options.

## Subjects and Methods

### Serum samples

Serum samples from HRMDD subjects and controls were collected from the Resource Center for Health Science (RECHS), Kyoto, Japan. RECHS is a biobank established to collect samples from healthy volunteers for precision medicine studies. The volunteers are recruited from subjects who come to a hospital for their annual health checkup. Once enrolled as a volunteer study participant at RECHS, the subject’s clinical data are collected and entered into a database, and serum samples are collected and stored in a −80 °C freezer every year. By utilizing the database and samples at RECHS, researchers can obtain control samples corresponding to interesting groups and series of samples collected over time.

To compare the HRMDD and healthy control groups, we selected 120 samples (61 HRMDD subjects and 59 age-matched healthy controls) from the RECHS database. The subjects in the HRMDD group were selected according to the following criteria: a CES-D score ≥16, a General Health Questionnaire 28 (GHQ-28)^44^ score ≥8, a positive score on the Brief Structured Interview for Depression (BSID)^45^ and no antidepressant use. The healthy controls were selected according to the following criteria: a CES-D score < 16, a GHQ-28 score < 8, and a negative BSID score. To ensure uniformity in terms of the clinical characteristics of the controls, the following criteria were also used: no current medications, a body mass index (BMI) < 25, intake of alcohol < 20 g/day, no smoking, blood pressure <140/90 mmHg, fasting blood sugar (FBS) < 110 mg/dL, low-density lipoprotein (LDL) < 140 mg/dL, high-density lipoprotein (HDL) > 40 mg/dL and triglyceride (TG) < 150 mg/dL.

To compare the changes in the levels of TRP metabolites and cytokines in subjects who progressed from a healthy state to a depressive state, we selected 99 samples (33 HRMDD subjects and 66 age-matched healthy controls) from the RECHS database. The target subjects who progressed from a healthy state to a depressed state were selected according to the following criteria: a CES-D score for the first test (CES-D_1st_) < 16, a CES-D score for the second test (CES-D_2nd_) ≥ 16 and a CES-D_2nd_ - CES-D_1st_ > 10. Two subjects were taking an antidepressant, and 3 subjects were taking a tranquilliser. The controls who had been in a healthy state for two years were selected according to the following criteria: CES-D scores for the first and second tests <8, no antidepressant use, no tranquilliser use, and no use of sleeping pills.

To compare the levels of TRP metabolites in chronic pain disorder patients and healthy controls, we collected samples from 48 chronic pain disorder patients and 42 healthy controls. Chronic pain disorder patients were recruited from the Liaison Outpatient Clinic of Aichi-Gakuin University Dental Hospital and were diagnosed with BMS and/or AO by dentists and pain disorder by psychiatrists according to the criteria in the diagnostic and statistical manual of mental disorders, 4th edition text revision (DSM-IV-TR)^46^. Among the chronic pain disorder patients, 4 subjects were diagnosed with MDD, and the mean ± standard deviation (SD) of the Hamilton Depression Rating Scale (HDRS)^47^ score was 6.69 ± 5.10. The 42 corresponding healthy controls who had no orofacial pain or psychiatric disorders were recruited from the same hospital and matched in terms of age and sex.

The study protocols were approved by the Ethics Committee of Kyoto University Graduate School of Medicine (authorization number R0544), Nagoya University Graduate School of Medicine (authorization number 2004-0234-2), and the Ethics Committee of the School of Dentistry, Aichi-Gakuin University (authorization number 372). All study protocols were performed according to the Declaration of Helsinki and the Ethical Guidelines for Epidemiological Research of the Ministry of Education, Culture, Sports, Science, and Technology and the Ministry of Health, Labour, and Welfare of Japan. Every effort was made to protect patient confidentiality and personal information. All participants, aged 20 years or older, provided written informed consent.

### TRP metabolite measurement

TRP, KYN, AA, KYNA and 3HAA were quantified by HPLC (SHIMAZU, Kyoto, Japan). Serum was mixed with 10% perchloric acid (4:1) and the mixture was centrifuged at 14000 rpm for 10 min (4 °C); then, 50 µl of supernatant was injected into the HPLC instrument for quantification. The mobile phase consisted of 10 mM sodium acid containing 1.7~2.0% acetonitrile, which was pumped at a flow rate of 0.8 ml/min. The analytical column was a TSKgel ODS-100v (TOSOH, Tokyo, Japan) that was 4.6 mm × 150 mm with a particle size of 3 µm and was kept at 30 °C. TRP and KYN were measured by a photodiode array detector (SPD-M30A: SHIMAZU, Kyoto, Japan) at wavelengths of 280 nm and 365 nm, respectively. AA, 3HAA and KYNA were measured by a fluorescence detector (RF-20Axs: SHIMAZU, Kyoto, Japan) at an excitation wavelength of 320 nm and an emission wavelength of 420 nm for AA and 3HAA and at an excitation wavelength of 334 nm and an emission wavelength of 380 nm for KYNA.

3HK was quantified by HPLC with an ECD 300 electrochemical detector (Eicom, Kyoto, Japan) with an applied potential of 550 mV. Serum was mixed with 10% perchloric acid (1:4), and the mixture was centrifuged at 14000 rpm for 10 min (4 °C); then, 20 µl of supernatant was injected into the HPLC instrument. The mobile phase consisted of 0.34 mM EDTA, 14 mM sodium heptane sulfonate, 5% phosphoric acid and 0.9% triethylamine containing 4% acetonitrile and was pumped at a flow rate of 0.5 ml/min. The column used was an EICOMPAK SC-5ODS (Eicom, Kyoto, Japan) that was 3.0 mm × 150 mm with a particle size of 5 µm and was kept at 25 °C.

An HPLC calibration curve for metabolites was examined. The value of the correlation coefficient (R2) was >0.99 within the tested concentration range (TRP: from 97.9 to 3.1 μM, KYN: from 3.84 to 0.1 μM, 3HAA: from 163.3 to 5.1 nM, KYNA: from 132.1 to 4.1 nM, AA: from 72.9 to 2.3 nM, and 3HK: from 446.1 to 3.4 nM). The limit of detection (LOD) is defined as the lowest concentration of metabolites in a sample that can be positively identified against the background. The limit of quantification (LOQ) is the lowest concentration of metabolites that can be accurately measured under the conditions of the method. The precision of the method was tested by performing ten independent intraday replicate measurements of 3 different concentrations of the standard. The SD and coefficient of variation {CV (%)} were calculated. All values were in the acceptable tested concentration range of the standard {CV (%) < 5}. Accuracy was determined by the recovery test. The recovery tests for the method showed that the HPLC assay was satisfactorily applicable for biological samples (95–102%)^15,48^.

### Cytokine and chemokine measurement

The levels of cytokines, including IL-1α, IL-1β, IL-1 receptor antagonist (IL-1RA), IL-2, IL-3, IL-4, IL-5, IL-6, IL-7, IL-8, IL-10, IL-12 (p40), IL-12 (p70), IL-13, IL-15, IL-17, interferon gamma-induced protein 10 (IP-10), granulocyte-macrophage-colony-stimulating factor (GM-CSF), granulocyte colony-stimulating factor (G-CSF), interferon-alpha 2 (IFN-α2), interferon-gamma (IFN-γ), monocyte chemoattractant protein-1 (MCP-1), macrophage inflammatory protein-1α (MIP-1α), macrophage inflammatory protein-1β (MIP-1β), normal T cell expressed and secreted (RANTES), vascular endothelial growth factor (VEGF), epidermal growth factor (EGF), eotaxin, tumour necrosis factor-α (TNF-α) and TNF-β, in the serum samples were measured with a MILLIPLEX multiplex assay system (Millipore, Billerica, MA) according to the manufacturer’s instructions. Twenty-five microliters of standard or serum sample was added to each well of the plate. Then, 25 μl of beads was added to each well and the plate was incubated overnight. Detection antibodies and streptavidin phycoerythrin were used for signal detection. Median fluorescence intensity data were analysed with Luminex200^TM^ software.

### Chronic unpredictable mild stress (CUMS) mouse model

The mice were randomly divided into control and CUMS groups. The mice were subjected to the CUMS procedure, as previously described^49^, for 4 weeks. Mice were subjected to the following kinds of stressors (one stressor per day): 12 h period of food and water deprivation, 12 h period of 30° tilted cage, 10 min period of cage shaking at 100 rpm, 12 h period of damp bedding in the cage, 1 min period of tail clamping, 2 h period of confinement in an air-permeable 50 ml tube, 24 h period of illumination (50 lx), 12 h period of empty cage and 24 h period of isolation. Each stressor was randomly repeated 3–4 times during the 4-week stress procedure.

### Social interaction test

The social interaction test was performed according to the method outlined in a previous report^50^. The apparatus used for the social interaction test consisted of an open square arena (30 × 30 × 35 cm) with no top, made of transparent acrylic and illuminated with lamps (that could not be seen by the mice directly). The light did not directly illuminate the arena and was diffused to minimize shadows in the arena (5 lx). Each mouse was placed alone in the test box for 10 min on 2 consecutive days before the social interaction test (habituation). On the test day, each mouse was randomly assigned to an unfamiliar partner. The unfamiliar partner was a sex-matched, 7-week-old C57BL/6 J mouse, that had been group housed and was naive to CUMS but not a cage mate. The mouse and the unfamiliar partner were placed in the box for 10 min.

### Statistics

The obtained values are expressed as the mean ± SD. To determine the correlations between the quantified parameters, Pearson’s product moment correlation coefficient and its statistical significance were calculated. Welch’s t test was used to compare the unpaired groups. Paired group comparisons were made with a paired t test. P-values less than 0.05 were considered statistically significant.

## Supplementary information

Dataset 1.
