# Supplementary material for: Serum Metabolic Profiles of the Tryptophan-Kynurenine Pathway in the high risk subjects of major depressive disorder
Source: Sci Rep. 2020 Feb 6;10:1961. doi: 10.1038/s41598-020-58806-w (PMC7005270; doi:10.1038/s41598-020-58806-w)
Supplement: Supplementary file 1 — Dataset 1. [file 41598_2020_58806_MOESM1_ESM.pdf]

Serum Metabolic Profiles of the Tryptophan-Kynurenine Pathway in the high risk subjects of major depressive disorder

Masashi Sakurai, Yasuko Yamamoto, Noriyo Kanayama, Masaya Hasegawa, Akihiro Mouri, Masao Takemura, Hidetoshi Matsunami, Tomoya Miyauchi, Tatsuya Tokura, Hiroyuki Kimura, Mikiko Ito, Eri Umemura, Aiji Sato (Boku), Wataru Nagashima, Takashi Tonoike, Kenichi Kurita, Norio Ozaki, Toshitaka Nabeshima and Kuniaki Saito

Inventory of Supplemental Information

Supplemental Figures (Figure S1)

Legends to Supplemental Figure S1

Supplemental Table 1-3

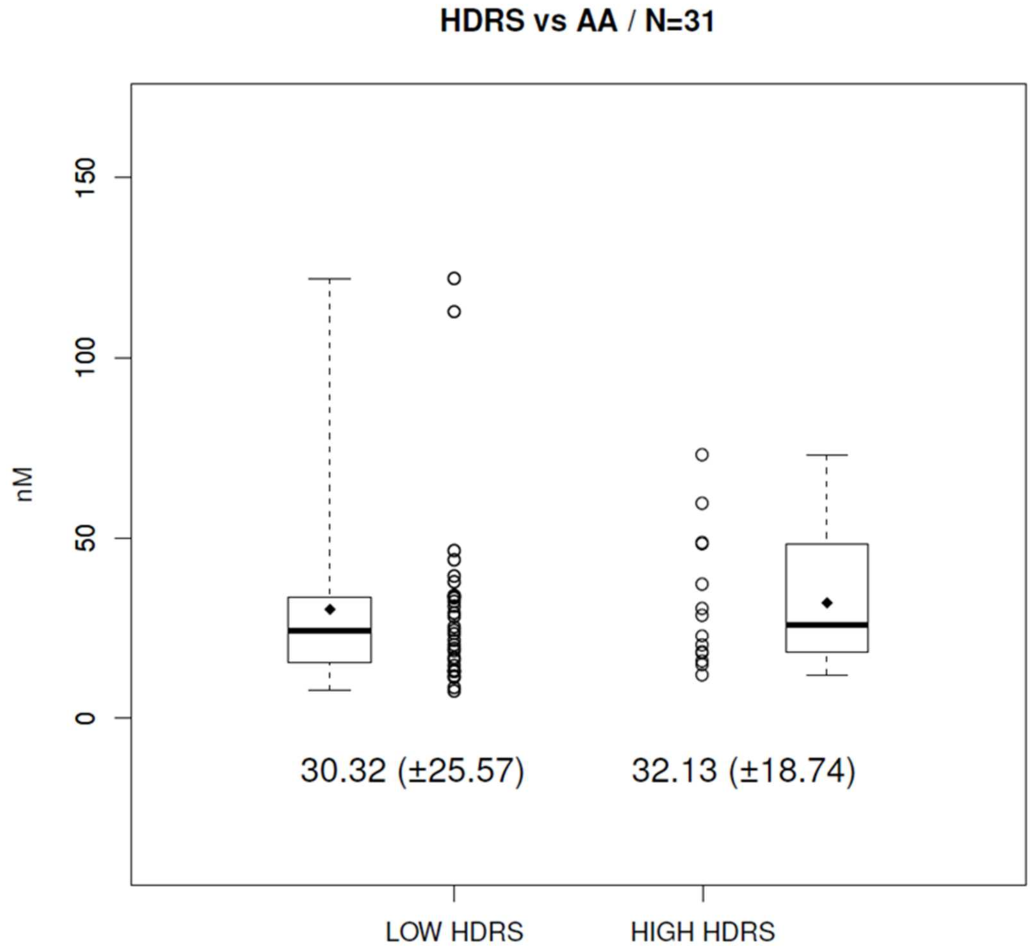

Supplemental Figure. 1

Supplemental Figure 1

Relationship between HDRS scores and AA levels.

The AA levels were compared between the low-HDRS group and the high-HDRS group.

High HDRS slightly increased the AA levels. However, the difference was not significant.

Supplemental Table 1: Concentrations of tryptophan metabolites and relative ratios of the metabolites in the healthy control and the subjects who progressed from a healthy state to a depressive state (high risk subjects of MDD, HRMDD)

	Healthy control		HRMDD	
	1st	2nd	1st	2nd
Trp (μM)	51.52 (7.29)	52.76 (7.77)	54.19 (8.51)	52.87 (7.59)
Kyn (μM)	1.62 (0.39)	1.62 (0.39)	1.56 (0.38)	1.54 (0.26)
KYNA (nM)	27.09 (9.81)	26.67 (9.21)	26.24 (8.15)	25.16 (8.01)
3HAA (nM)	14.52 (5.40)	14.19 (4.96)	14.89 (5.01)	15.42 (7.15)
AA (nM)	12.81 (9.56)	11.17 (4.32)	10.54 (3.56)	11.58 (4.54)
3HK (nM)	81.00 (39.18)	66.81 (28.24)*	83.47 (35.72)	77.84 (35.26)
Kyn/Trp	0.032 (0.007)	0.031 (0.007)	0.029 (0.008)	0.030 (0.006)
KYNA/Kyn	16.94 (5.79)	16.84 (5.92)	17.07 (4.42)	16.41 (4.49)
AA/Kyn	8.19 (6.84)	7.30 (4.13)	7.03 (2.37)	7.76 (3.48)
3HK/Kyn	51.07 (22.87)	43.31 (21.68)*	56.94 (31.79)	52.45 (27.40)
3HAA/3HK	0.21 (0.09)	0.24 (0.12)	0.21 (0.09)	0.24 (0.13)
3HAA/AA	1.32 (0.59)	1.41 (0.60)	1.56 (0.73)	1.49 (0.75)

Supplemental Table 2: Concentrations of cytokines in the subjects who progressed from a healthy state to a depressive state (high risk subjects of MDD, HRMDD)

	HRMDD	
	1st	2nd
IL-1α	45.91 (36.91)	53.52 (44.44)
IL-1β	4.70 (2.87)	3.77 (2.82)
IL-1RA	37.53 (22.28)	36.70 (19.63)
IL-2	7.80 (5.41)	5.20 (3.90) **
IL-3	3.24 (5.26)	1.86 (4.33)
IL-4	21.13 (31.56)	24.76 (30.99)
IL-5	1.17 (2.01)	1.91 (2.31)
IL-6	5.77 (10.70)	6.93 (12.18)
IL-7	47.45 (13.44)	47.50 (13.54)
IL-8	48.66 (21.07)	46.41 (20.07)
IL-10	11.34 (11.76)	10.29 (7.92)
IL-12(P40)	28.57 (18.50)	22.75 (21.53)
IL-12(P70)	15.07 (16.16)	12.82 (17.57)
IL-13	3.78 (6.65)	3.51 (5.83)
IL-15	16.40 (7.61)	16.82 (9.71)
IL-17	15.24 (11.27)	15.01 (10.53)
IP-10	410.23 (146.59)	413.48 (129.80)
GM-CSF	11.29 (12.16)	13.21 (14.82)
G-CSF	101.61 (69.47)	90.75 (76.21)
IFN-α2	71.72 (47.34)	85.53 (46.55)
IFN-γ	27.26 (8.79)	22.20 (10.07) *
MCP-1	1291.29 (343.73)	1265.35 (349.25)
MIP-1α	32.61 (23.51)	31.05 (20.59)
MIP-1β	143.38 (86.55)	137.63 (78.58)
RANTES	4738.62 (3666.78)	5516.24 (3819.17)
VEGF	410.88 (740.68)	465.60 (1038.56)
EGF	199.24 (158.30)	182.67 (150.00)
EOTAXIN	328.91 (114.07)	335.28 (127.17)
TNF-α	90.34 (29.41)	87.18 (25.37)
TNF-β	10.28 (12.81)	10.31 (15.15)

The obtained values are expressed as the mean±SD. *P<0.05, **P<0.01; significant difference in values between first and second tests as determined by paired t test.

Supplemental Table 3: Correlation coefficients between cytokine and ΔCES-D or AA

	Cytokine	Correlation coefficient	p value
ΔCES-D	IL-15	0.258	0.161
	IFN-γ	0.226	0.221
	EOTAXIN	0.203	0.273
	IL-6	0.202	0.276
	G-CSF	-0.233	0.207
AA	IL-12(P70)	0.374	0.038
	IL-5	0.309	0.090
	TNF-β	-0.237	0.200
	GM-CSF	-0.252	0.172
	IP-10	-0.324	0.076
	MCP-1	-0.398	0.027
	G-CSF	-0.431	0.016
	IL-1RA	-0.556	0.001

The correlation was determined and the Pearson's product moment correlation coefficient and the significance of the correlation (P) are shown.
